# Transient secondary pseudo-hypoaldosteronism in infants with urinary tract infections: systematic literature review

**DOI:** 10.1007/s00431-024-05676-3

**Published:** 2024-07-10

**Authors:** Céline Betti, Camilla Lavagno, Mario G. Bianchetti, Lisa Kottanattu, Sebastiano A. G. Lava, Federica Schera, Marirosa Cristallo Lacalamita, Gregorio P. Milani

**Affiliations:** 1 Pediatric Institute of Southern Switzerland, Ente Ospedaliero Cantonale, Bellinzona, Switzerland; 2 Faculty of Biomedical Sciences, Università Della Svizzera Italiana, Lugano, Switzerland; 3 Pediatric Emergency Department, University Children’s Hospital Zurich, Zurich, Switzerland; 4 Family Medicine, Faculty of Biomedical Sciences, Università Della Svizzera Italiana, Lugano, Switzerland; 5 Pediatric Cardiology Unit, Department of Pediatrics, Centre Hospitalier Universitaire Vaudois and University of Lausanne, Lausanne, Switzerland; 6 Clinical Pharmacology Service, Centre Hospitalier Universitaire Vaudois and University of Lausanne, Lausanne, Switzerland; 7 Imaging Institute of Southern Switzerland, Ente Ospedaliero Cantonale, Bellinzona, Switzerland; 8 Pediatric Unit, Fondazione IRCCS Ca’ Granda Ospedale Maggiore Policlinico, Via Della Commenda 9, 20122 Milan, Italy; 9 Department of Clinical Sciences and Community Health, Università Degli Studi Di Milano, Milan, Italy

**Keywords:** Acidosis, Hyperkalemia, Hyponatremia, Under-responsiveness to aldosterone, Urinary tract infection

## Abstract

Infants with a congenital anomaly of the kidney and urinary tract sometimes present with hyponatremia, hyperkalemia, and metabolic acidosis due to under-responsiveness to aldosterone, hereafter referred to as secondary pseudo-hypoaldosteronism. The purpose of this report is to investigate pseudo-hypoaldosteronism in infant urinary tract infection. A systematic review was conducted following PRISMA guidelines after PROSPERO (CRD42022364210) registration. The National Library of Medicine, Excerpta Medica, Web of Science, and Google Scholar without limitations were used. Inclusion criteria involved pediatric cases with documented overt pseudo-hypoaldosteronism linked to urinary tract infection. Data extraction included demographics, clinical features, laboratory parameters, management, and course. Fifty-seven reports were selected, detailing 124 cases: 95 boys and 29 girls, 10 months or less of age (80% of cases were 4 months or less of age). The cases exhibited hyponatremia, hyperkalemia, acidosis, and activated renin-angiotensin II-aldosterone system. An impaired kidney function was found in approximately every third case. Management included antibiotics, fluids, and, occasionally, emergency treatment of hyperkalemia, hyponatremia, or acidosis. The recovery time averaged 1 week for electrolyte, acid–base imbalance, and kidney function. Notably, anomalies of the kidney and urinary tract were identified in 105 (85%) cases.

*Conclusions*:This review expands the understanding of overt transient pseudo-hypoaldosteronism complicating urinary tract infection. Management involves antimicrobials, fluid replacement, and consideration of electrolyte imbalances. Raising awareness of this condition within pediatric hospitalists is desirable.

**Table:** 

**What is Known**:
• *Infants affected by a congenital anomaly of the kidney and urinary tract may present with clinical and laboratory features resembling primary pseudo-hypoaldosteronism.* • *Identical features occasionally occur in infant urinary tract infection.*
**What is New**:
• *Most cases of secondary pseudo-hypoaldosteronism associated with a urinary tract infection are concurrently affected by a congenital anomaly of the kidney and urinary tract.* • *Treatment with antibiotics and parenteral fluids typically results in the normalization of sodium, potassium, bicarbonate, and creatinine within approximately 1 week.*

• *Infants affected by a congenital anomaly of the kidney and urinary tract may present with clinical and laboratory features resembling primary pseudo-hypoaldosteronism.*

• *Identical features occasionally occur in infant urinary tract infection.*

• *Most cases of secondary pseudo-hypoaldosteronism associated with a urinary tract infection are concurrently affected by a congenital anomaly of the kidney and urinary tract.*

• *Treatment with antibiotics and parenteral fluids typically results in the normalization of sodium, potassium, bicarbonate, and creatinine within approximately 1 week.*

## Introduction

Primary pseudo-hypoaldosteronism is a rare autosomal hereditary disorder marked by resistance to aldosterone, leading to renal salt wasting, hypovolemia, tendency to low blood pressure, hyponatremia, hyperkalemia, metabolic acidosis, and elevated renin and aldosterone levels [1, 2]. There is a recessive form, which affects all aldosterone target organs and is permanent, and a dominant (or sporadic) form, which is limited to the kidney and often improves with age [1, 2]. Features resembling primary pseudo-hypoaldosteronism can also occur in infants with congenital, mostly obstructive, anomalies of the kidney and urinary tract [3–5]. The latter condition, initially documented in the 1980s by J. Rodríguez-Soriano (1933–2010), is termed overt transient secondary pseudo-hypoaldosteronism and will be hereafter referred to as secondary pseudo-hypoaldosteronism [3].

Secondary pseudo-hypoaldosteronism may also complicate severe acute urinary tract infections [4, 5]. This condition has been rarely documented. Consequently, comprehensive evidence is scarce regarding the underlying mechanisms, its association with anomalies of the kidney and urinary tract, the most effective diagnostic and management strategies, and the duration of recovery. To address these issues, we undertook a systematic review of the literature.

## Methods

### Literature search strategy

This study was pre-registered with the International Prospective Register of Systematic Reviews (PROSPERO: CRD42022364210) and conducted following the guidelines of the 2020 edition of the Preferred Reporting Items for Systematic Reviews and Meta-Analyses (PRISMA) methodology [6]. The search for relevant literature was carried out in the National Library of Medicine, Excerpta Medica, and Web of Science databases, with no restrictions on date or language. Search terms were “urinary tract infection” AND “pseudo-hypoaldosteronism” OR “aldosterone” OR “hyponatremia” OR “acidosis” OR “hyperkalemia.” References listed within bibliographies of the retrieved records, reports already known to the authors, and Google Scholar were also considered for inclusion [7]. The search was conducted in October 2023 and repeated before submission (April 15, 2024). Following a preliminary selection round based on title and abstract, the full text of the selected reports was assessed for eligibility.

### Selection criteria—data extraction

Original articles reporting individually documented cases of secondary pseudo-hypoaldosteronism temporally associated with a urinary tract infection were sorted. Included were pediatric patients with a urinary tract infection and at least three of the following four biochemical laboratory abnormalities [5]: hyponatremia (sodium < 135 mmol/L); hyperkalemia (potassium > 5.4 mmol/L); non-anion gap metabolic acidosis (bicarbonate < 20 mmol/L and pH < 7.35); increased aldosterone level (standard deviation score > 2.0). The following seven variables were extracted: (1) demographics; (2) history of premature delivery or a pre-existing anomaly of the kidney and urinary tract; (3) clinical and laboratory data at presentation with emphasis on blood sodium, potassium, acid–base balance, calcium [8], creatinine, urea, aldosterone, and renin; (4) management including pre-existing treatment with drugs that may lead to hyponatremia, hyperkalemia, or acidosis such as trimethoprim, potassium-sparing diuretics, or blockers of the renin–angiotensin–aldosterone system; (5) the course of laboratory values; and (6) results of imaging studies [9] and surgical management. The possible clinical relevance of detected anomalies of the kidney and urinary tract was categorized as high, low, or unknown during an ad-hoc consensus conference among the authors.

Age-specific upper limit references for blood creatinine were used to classify acute kidney injury as stages 1, 2, or 3 using the KDIGO criteria [10, 11]. In subjects with a raised urea blood level, a molar urea-to-creatinine ratio ≥ 80 was considered a marker of fluid volume depletion [12].

To acquire missing data, attempts were also made to contact original authors. Two authors carried out the literature search in duplicate, selected the reports retained for analysis, and extracted the data. Discrepancies were solved by consensus and, where necessary, a third author was involved. One author entered the data into a pilot-tested database, and the second author verified the correctness of the data entry.

### Accuracy of reporting—analysis

The accuracy in reporting the six specified variables was assessed for each case on a scale of 0, 1, or 2. The reporting comprehensiveness was subsequently categorized based on the cumulative score of these factors, with classifications of excellent (≥ 10), good (7 to 9), or satisfactory (4 to 6). Missing data were handled by pairwise deletion. Sodium, potassium, bicarbonate, creatinine, and urea are expressed both as concentration and as standard deviation scores based on reference values. Aldosterone and renin were expressed uniquely as standard deviation scores. Categorical data are presented as counts and were analyzed using the Fisher’s exact test. Continuous data are shown as median and interquartile range or as box-and-whisker plot (the lower and upper boundaries of the box indicate the 25th and 75th centiles, respectively, and the central line within the box corresponds to the median, while the extremities of the whiskers represent the 3rd and 97th centiles) and were compared using the Kruskal–Wallis H-test. Regressions were conducted using the Spearman rank correlation test. Statistical significance was defined by two-sided *P*-values of < 0.05. GraphPad Prism version 10.2.3 (GraphPad Software, San Diego, CA, USA) was employed for statistical analysis.

## Results

### Search outputs—completeness of reporting

**Fig. 1:**
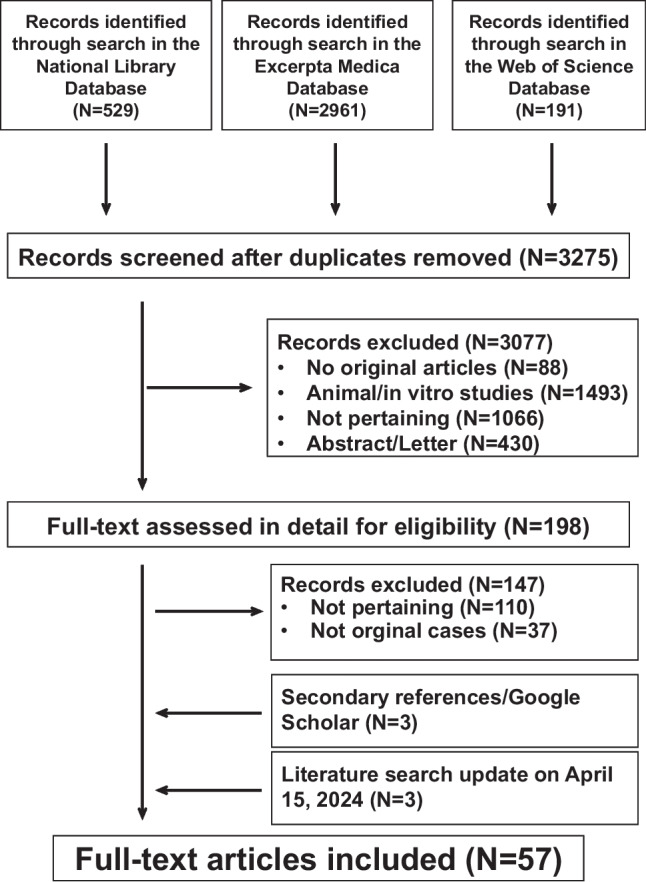
Secondary pseudo-hypoaldosteronism in children with a urinary tract infection. Flowchart of the literature search

The literature search returned 3275 potentially relevant reports (Fig. 1). After removing irrelevant reports, 198 full-text publications were reviewed for eligibility. For the final analysis, we retained 57 reports describing 124 individual cases of infant urinary tract infection complicated by secondary pseudo-hypoaldosteronism [3, 13–68]. The mentioned reports were published since 1983 in English (*N* = 51), Spanish (*N* = 3), Italian (*N* = 2), and Dutch (*N* = 1). Europe contributed 24, Asia 20, and America 13 reports. Reporting comprehensiveness was excellent in 21, good in 96, and satisfactory in the remaining 7 cases.

### Presentation

**Fig. 2:**
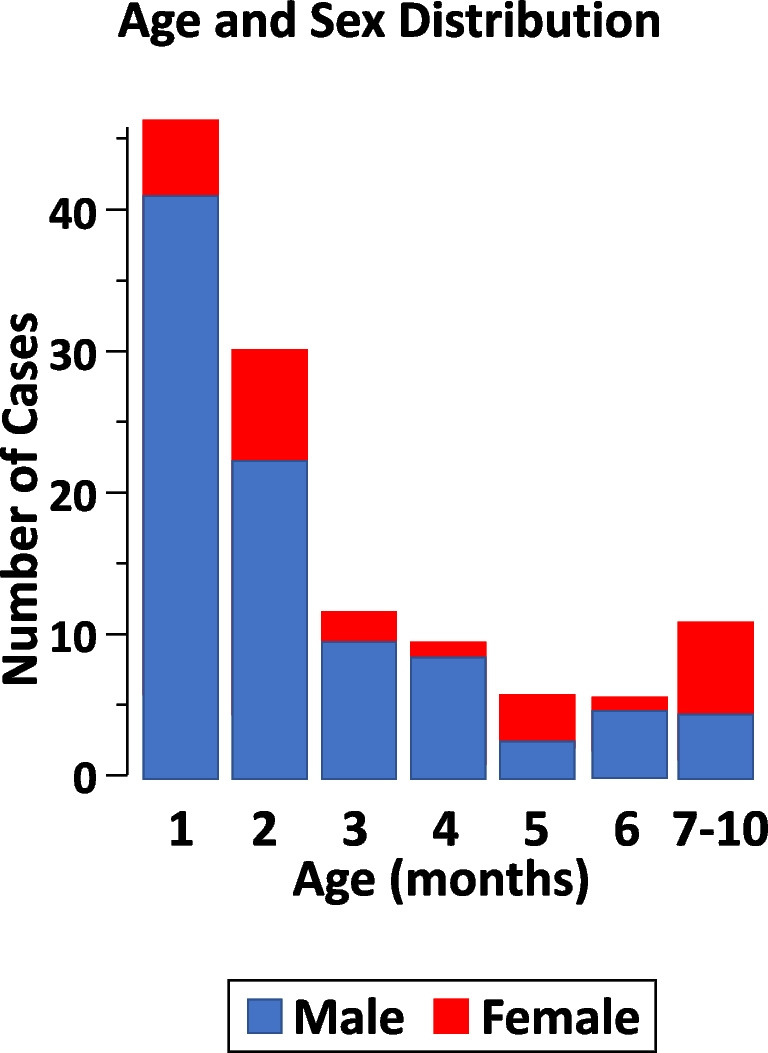
Age and sex distribution in 124 infants (95 boys and 29 girls) affected by pseudo-hypoaldosteronism secondary to a urinary tract infection

The age of the 124 patients (95 boys and 29 girls) is depicted in Fig. 2. Eighty percent (*N* = 99) of the cases were 4 months or less of age. Furthermore, none of them was more than 10 months of age. Three infants affected by a congenital anomaly of the kidney and urinary tract were on antimicrobial prophylaxis with low-dose (0.3 mg/kg body weight daily) trimethoprim.

**Fig. 3:**
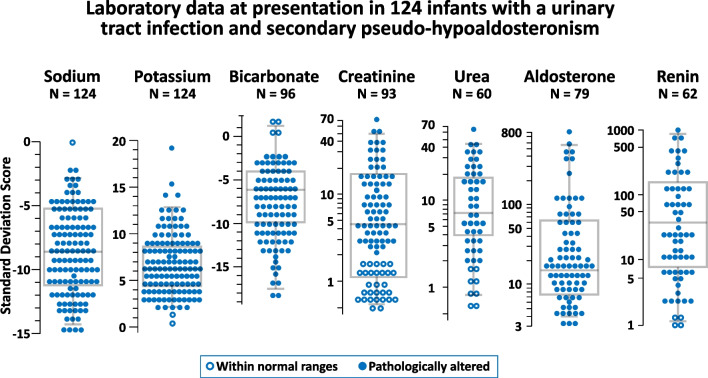
Standard deviation scores for sodium, potassium, bicarbonate, creatinine, urea, aldosterone, and renin in infants affected by pseudo-hypoaldosteronism secondary to a urinary tract infection. A logarithmic scale was chosen for creatinine, urea, aldosterone, and renin. Data are presented both as individual values and as boxplots (the lower and upper boundaries of the box signify the 25th and 75th percentiles, respectively; the central line within the box represents the median, while the extremities of the whiskers indicate the 3rd and 97th percentiles). Open symbols represent a normal, while filled symbols indicate a pathologically altered result

The main biochemical laboratory data are given in Table 1 and Fig. 3. Hyponatremia was detected in 99%, hyperkalemia in 98%, metabolic acidosis in 96%, increased creatinine in 69%, increased urea in 87%, hyperaldosteronism in all, and hyperreninemia in 93% of the cases with the corresponding laboratory measurement. Acute kidney injury was identified in 41 (33%) cases. The molar urea-to-creatinine ratio in blood, calculated in 52 patients with a raised urea blood level, was ≥ 80 in 50 cases. No significant correlation was found between aldosterone SDS, taken as an independent variable, and sodium SDS (*r*_s_ = 0.1753; *P* = 0.1881), potassium SDS (*r*_s_ = 0.1838; *P* = 0.1672), or bicarbonate SDS (*r*_s_ = 0.0668; *P* = 0.613), taken as dependent variables.

**Table 1:** Baseline characteristics of 124 infants affected by a severe urinary tract infection complicated by secondary pseudo-hypoaldosteronism. Data are presented either as frequency (often with percentage) or as median (with interquartile range)

*N*	124
Males:females, *N* (%)	95 (77):29 (23)
Age, months	1.5 (1.0–3.5)
History of premature delivery, *N* (%)	3 (2.4)
Prenatally suspected uropathy, *N* (%)	21 (17)
Low-dose trimethoprim prophylaxis, *N* (%)	3 (2.4)
Blood electrolytes	
Sodium, mmol/L	119 (113–125)
Potassium, mmol/L	6.8 (6.2–7.8)
Bicarbonate^*^, mmol/L	16 (12–17)
Kidney function	
Creatinine^✢^, µmol/L	47 (30–86)
Acute kidney injury, *N* (%)	41
Stage 1, *N*	11
Stage 2, *N*	13
Stage 3, *N*	17
Urea^◇^, mmol/L	14.2 (9.6–27.3)
Molar urea-to-creatinine ratio^▲^	
Value	274 (167–411)
≥ 80, *N* (%)	50
Isolated pathogen^†^	
*Escherichia coli*, *N*	54
*Klebsiella* species, *N*	18
*Enterococcus* species, *N*	11
Group-B streptococcus, *N*	6
*Proteus* species, *N*	4
*Pseudomonas* species, *N*	4
Further pathogens^‡^, *N*	9

^*^Information not available in 28 cases; ^✢^information not available in 31 cases; ^◇^information not available in 64 cases; ▲calculated uniquely in 52 patients with a raised urea blood level; †no pathogen reported in 18 cases; ^‡^*Staphylococcus aureus* (*N* = 3), *Enterobacter cloacae* (*N* = 2), *Serratia marcescens* (*N* = 2), *Morganella species* (*N* = 1), *Candida albicans* (*N* = 1)

Information on blood calcium levels was provided only in three cases [21, 37, 59]. Hypercalcemia (total calcium 2.97 mmol; upper reference 2.70) was noted in one of the three cases [59].

### Medical management—course

Antibiotics and fluids were prescribed in all cases. Other drugs were prescribed in 69 (56%) cases, as shown in Table 2. A disturbance of the renin–angiotensin–aldosterone system, mostly congenital adrenal hyperplasia, was suspected in approximately one out of every four cases. Consequently, in these individuals, steroids were administered in a dosage recommended for the adrenal salt-wasting crisis while awaiting adrenal test results.

**Table 2:** Non-antibiotic and non-fluid acute treatment in 69 infants (50 boys and 19 girls, aged between 0.3 and 10 months, with a median age of 1.4 months) diagnosed with a urinary tract infection and secondary pseudo-hypoaldosteronism. The apparent discrepancy between the patient count and the number of medications administered arises from the fact that some infants received multiple prescriptions. The drugs are listed in descending order of frequency of use, without any emphasis for their potential impact

Steroids, *N*	34
Hydrocortisone*, *N*	13
Fludrocortisone^◇^, *N*	4
Corticosteroids, *N*	3
Hydrocortisone* and fludrocortisone^◇^, *N*	14
Sodium bicarbonate, *N*	24
Loop diuretics, *N*	13
Calcium, *N*	8
Insulin, *N*	7
Cation-exchange resins, *N*	6
Hypertonic saline, *N*	5
β_2_-adrenergic agonists, *N*	3

^*^50–100 mg/m^2^ per day; ^◇^100–200 µg per day

Insulin, a β_2_-adrenergic agonist, a cation-exchange resin, or a diuretic were used to correct hyperkalemia in 29 cases.

Intravenous calcium salts were used in eight cases [21, 41, 45, 50, 54, 60]. Calcium was administered to prevent or manage cardiac arrhythmias in seven out of the eight cases (6 boys and 1 girl; age 1.8 (1.0–2.5) months). The cardiac arrhythmias included one case each of ventricular tachycardia, ventricular flutter, ventricular fibrillation, and cardiopulmonary arrest. Calcium was also administered to a 2-month-old female infant in circulatory shock.

Bicarbonate to fix acidosis and hypertonic saline to treat hypernatremia were also used in 24 and 5 cases, respectively.

The normalization of blood sodium, potassium, and bicarbonate levels was documented in 103, 98, and 69 cases. The normalization of creatinine levels was documented in 64 cases (including 42 cases with an acute kidney injury). The time to achieve a normal blood level was on average the same for sodium (7 (2–10) days; *N* = 65), potassium (7 (2–11) days; *N* = 55), bicarbonate (7 (3–14) days; *N* = 40), and creatinine (7 (3–8) days, *N* = 30). The figures did not statistically differ between infants given steroids and those without (Table 3). In 25 cases (19 boys and 6 girls 2.2 (1.2–3.9) months of age), a second determination of aldosterone level was performed 2.0 (1.6–5.7) weeks later. This parameter was found to decrease by 5.7 (1.8–12) SDS per week.

**Table 3:** Baseline characteristics of patients with and without steroid treatment, and time to achieve a normal sodium, potassium, bicarbonate, and creatinine level in blood. Data are presented either as frequency (with percentage) or as median (with interquartile range)

	Steroids		*P*-values
	With	Without	
Cases, *N*	34	90	
Males:females, *N* (%)	27: 7	68: 22	0.8129
Age months	1.5 (1.0–3.1)	1.9 (1.0–3.9)	0.4993
Baseline blood values			
Sodium, mmol/L	116 (110–120)	121 (114–128)	0.0005
Potassium, mmol/L	7.3 (6.5–8.4)	6.7 (6.1–7.4)	0.0252
Bicarbonate^*^, mmol/L	15 (13–18)	16 (12–18)	0.7793
Acute kidney, injury, *N*	12	29	0.8312
Time to normalize*, days			
Sodium	5 (2–9)	7 (3–13)	0.3010
Potassium	6 (2–9)	7 (2–14)	0.4408
Bicarbonate	5 (2–10)	7 (4–24)	0.376
Creatinine	8 (2–9)	7 (4–8)	0.8588

^*^Documented uniquely in a minority of cases (see text)

Among the 124 infants, there were no reported fatalities.

### Anomalies of the kidney and urinary tract—surgery

Anomalies of the kidney and urinary tract (Table 4) were identified in 105 (85%) cases (including 21 already prenatally suspected uropathies) but were often clinically not relevant. A surgical repair was undertaken in 52 of the 105 cases. Age (*P* = 0.9204) and male to female ratio (*P* = 0.1034) were not statistically different in subjects without a urinary tract anomaly (1.5 (1.1–2.3) months of age; 17 boys and 2 girls), in subjects with urinary tract anomalies not undergoing surgery (2.0 (1.1–2.9) months; 35 boys and 18 girls), and in subjects with urinary tract malformations undergoing surgical repair (2.1 (0.9–3.1) months; 43 boys and 9 girls).

**Table 4:** Anomalies of the kidney and urinary tract identified in 105 infants (78 boys and 27 girls; 0.2 to 10, median 1.5 months of age) with a urinary tract infection complicated by secondary pseudo-hypoaldosteronism. One anomaly was detected in 97 and two in the remaining 8 cases. The currently suggested terminology for nephro-urological radiology was used [9]

Anomaly	*N*	Relevance
Vesicoureteral reflux^†^	51	
High grade (≥ III)	37	High
Low grade (I, II)	4	Low
Unspecified	10	Unknown
Posterior urethral valves	15	High
Pelvic and ureteral duplication	14	
Unilateral	13	Low
Bilateral	1	Low
Pelvi-ureteral junction obstruction	12	High
Obstructive ureteral dilatation	7	High
Unilateral ectopic ureteral insertion	3	Low
Renal dysplasia	3	Low
Unilateral renal hypoplasia	2	Low
Solitary kidney	2	Low
Others*	4	Low

^†^In cases with bilateral reflux (*N* = 21), the highest grade is reported; *unilateral mild atrophy (*N* = 1), atrophic unilateral upper pole (*N* = 1), unilateral multicystic dysplastic kidney (*N* = 1), persistent urogenital sinus (*N* = 1)

## Discussion

Available knowledge on secondary pseudo-hypoaldosteronism is mainly based on case reports and very limited case series. The results of this systematic review of the literature on secondary pseudo-hypoaldosteronism in infant urinary tract infection may be summarized as follows: (1) Approximately 80% of affected patients are boys (with a history of term birth) 4 months or less of age; (2) the presentation includes fluid volume depletion, hyponatremia, hyperkalemia, metabolic acidosis, kidney function impairment, and an extremely activated renin-angiotensin II-aldosterone system; (3) hyperkalemia occasionally results in life-threatening cardiac arrhythmias; (4) a surgically relevant urinary tract malformation is detected in at least 40% of cases; (5) electrolytes, acid–base imbalance, and kidney function normalize on average 1 week after appropriated therapy, and this process is not accelerated by steroid therapy; (6) the activation of the renin-angiotensin II-aldosterone system might persist longer than the electrolyte and acid–base imbalance; (7) despite occasional severe cardiac arrhythmias induced by hyperkalemia, no fatalities were documented.

Initially identified in the 1980s, secondary pseudo-hypoaldosteronism was first observed in infants with obstructive uropathy, either with or without a concurrent urinary tract infection [2–4]. Some preliminary data link need for surgery with renin-aldosterone system activity [69]. The findings of this review provide evidence supporting the involvement of kidney inflammation in this condition. The causes of aldosterone under-responsiveness during infant urinary tract infection remain unclear. However, three main explanations have been proposed: (a) it may be triggered by an inflammatory storm, partly mediated by transforming growth factor-β [1, 70, 71]; (b) differences in kidney morphology and function between infants and older children might also contribute [3, 72, 73]; (c) genetic factors like those seen in dominant primary pseudo-hypoaldosteronism might occasionally be present in secondary cases [2, 60].

In infants with secondary pseudo-hypoaldosteronism and a urinary tract infection, kidney damage may arise from (a) the direct impact of the acute urinary infection on kidney tissue; (b) fluid volume depletion due to aldosterone under-responsiveness, leading to reduced kidney perfusion; and (c) pre-existing anomalies in the kidney and urinary tract, though this is unlikely here since creatinine levels normalized in all cases. In the pediatric general population, a severe urinary tract infection leads to acute kidney injury in roughly 15% of instances [74]. In secondary pseudo-hypoaldosteronism, these figures rise to around 30%. Fluid volume depletion resulting from aldosterone under-responsiveness likely accounts for the difference.

Adrenal insufficiency and primary pseudo-hypoaldosteronism are rare but recognized causes of hypercalcemia [8]. In secondary pseudo-hypoaldosteronism, calcium levels were elevated in one out of three cases examined.

The prevalence of overt secondary pseudo-hypoaldosteronism is unknown but likely very low. Less overt cases present with mild electrolyte or acid–base imbalances and likely occur in one out of five infants with a severe urinary tract infection [64]. Additionally, cases with normal sodium, potassium, and bicarbonate levels but with increased aldosterone and renin levels have been documented [70, 71, 75].

In infant urinary tract infection complicated by secondary pseudo-hypoaldosteronism, the cornerstone of acute management includes administering high-dose, broad-spectrum antibiotics with high tissue penetration [76] and replacing fluids using an isotonic solution [3, 5, 70, 75]. It is also recommended to consider discontinuing medications associated with hyponatremia, hyperkalemia, or acidosis [77, 78]. The assessment of fluid volume depletion via history and examination is notoriously challenging in individuals with hyponatremia [79, 80]. It is generally accepted that the urea-to-creatinine ratio and the electrolyte disturbances in blood reliably indicate the degree of depletion [12, 80]. Normal saline is preferred to restore hyponatremia and lactated Ringer to correct acidosis [81]. Hyperkalemia habitually contraindicates Ringer because it contains potassium. However, this view is not supported by the literature [5, 81].

Hyponatremia may be severe (≤ 120 mmol/L) in secondary pseudo-hypoaldosteronism. Since hyponatremia develops over ≥ 48 h, a rapid repair risks brain damage. Most authorities currently advise to aim for a ≤ 6–8 mmol/L daily increase in sodium [82, 83]. Both in primary and secondary pseudo-hypoaldosteronism, isotonic solutions correct not only volume depletion, hyponatremia, and acidosis but also hyperkalemia [1–3, 5, 70]. Mostly, it is not necessary to use β_2_-adrenergic agonists, bicarbonate, insulin, or cation-exchange resins to correct hyperkalemia or calcium to counteract the effects of hyperkalemia on cardiac cell membranes [84]. This is also in consideration of the possible side effects [84]. Finally, the present analysis shows that, like in primary pseudo-hypoaldosteronism [1, 2], hydrocortisone and fludrocortisone are not indicated.

This analysis exhibits both limitations and strengths. The rarity of secondary pseudo-hypoaldosteronism results in a limited sample size and affects the study’s generalizability. Additionally, there are examples of incomplete reporting, such as the omission of urine output in the diagnosis of this type of community-acquired acute kidney injury. A further limitation of the study arises from inconsistent laboratory techniques used for the measurement of sodium, creatinine, renin, and aldosterone. On the other hand, the study is registered and conducted in accordance with a recognized methodology [6]. Furthermore, the study’s comprehensive approach involved the utilization of four databases [7]. Finally, collaboration with different pediatric specialists enhances its robustness.

## Conclusions

The link between acute urinary tract infection and aldosterone under-responsiveness is considered rare. Pediatric hospitalists confidently diagnose this condition through additional tests like renin, aldosterone, cortisol, and 17-hydroxyprogesterone, with results typically taking several days [8, 54, 85]. Salt-wasting crises in congenital adrenal hyperplasia can mimic pseudo-hypoaldosteronism, but due to the implementation of neonatal screening, such cases are now uncommon in many countries [85]. Hence, most cases presenting with hyponatremia, hyperkalemia, and metabolic acidosis in infancy are currently associated with a urinary tract infection or a urinary tract anomaly. Raising awareness of the condition is desirable.

## Data Availability

Data sharing does not apply to this report since no new data were generated during this study.

## Declarations

### Generative artificial intelligence

The authors declare that they used generative artificial intelligence to improve the readability and language of the manuscript.
